# Platelets in systemic lupus erythematosus: from hemostatic allies to pathogenic drivers

**DOI:** 10.3389/fimmu.2026.1778274

**Published:** 2026-07-24

**Authors:** Yanzuo Wu, Ze Yang, Shuo Huang, Jie Bao, Yongsheng Fan, Guanqun Xie

**Affiliations:** 1 The First School of Clinical Medicine, Zhejiang Chinese Medical University, Hangzhou, Zhejiang, China; 2 School of Basic Medical Sciences, Zhejiang Chinese Medical University, Hangzhou, Zhejiang, China; 3 Department of Rheumatology, The Second Affiliated Hospital of Zhejiang Chinese Medical University, Hangzhou, Zhejiang, China

**Keywords:** inflammation, lupus nephritis, platelet, platelet-derived microparticles, systemic lupus erythematosus

## Abstract

Systemic lupus erythematosus (SLE) is a multisystem autoimmune disease; hematologic involvement is common and strongly associated with prognosis. Platelets—the second most abundant cellular component of peripheral blood—are anucleate cytoplasmic fragments. Their canonical roles include hemostasis, thrombosis, and vascular repair. More recently, platelets have emerged as regulators of innate and adaptive immunity that amplify inflammatory signaling and may facilitate tumor dissemination, suggesting underappreciated pathogenic roles in the initiation and progression of SLE. This review synthesizes platelet pathobiology in SLE and structures it into four modules: (i) pro-inflammatory actions of activated platelets; (ii) pathogenic effects of platelet-derived microparticles (PMPs); (iii) mechanisms by which platelets exacerbate lupus nephritis; and (iv) pathways linking platelets to SLE-associated cardiovascular disease. We also evaluate platelet-targeted therapeutic strategies and their translational prospects. Our aim is to provide a coherent framework for the platelet–SLE interface that informs mechanistic studies, guides biomarker development, and supports the design of more precise diagnostics and therapies.

## 1 Introduction

Systemic lupus erythematosus (SLE) is a chronic autoimmune disorder that affects multiple organ systems and arises from a complex dysregulation of the immune response (1, 2). Central to its pathology is the loss of immune tolerance to self-antigens, leading to the formation and deposition of immune complexes. These complexes trigger persistent inflammation and contribute to multi-organ damage (3–6). Circulating abnormal immune cells further amplify disease activity by releasing pro-inflammatory mediators and promoting vascular injury. Platelets, the second most abundant cellular component of blood after erythrocytes, are anucleate fragments traditionally recognized for their roles in hemostasis, coagulation, and vascular repair (7, 8). When blood vessels are injured, platelets rapidly aggregate at the site of damage to form an initial plug that prevents blood loss. Simultaneously, they trigger the coagulation cascade by releasing clotting and platelet-derived factors, ultimately generating a stable fibrin network to complete hemostasis (9–12).

**Figure 1:**
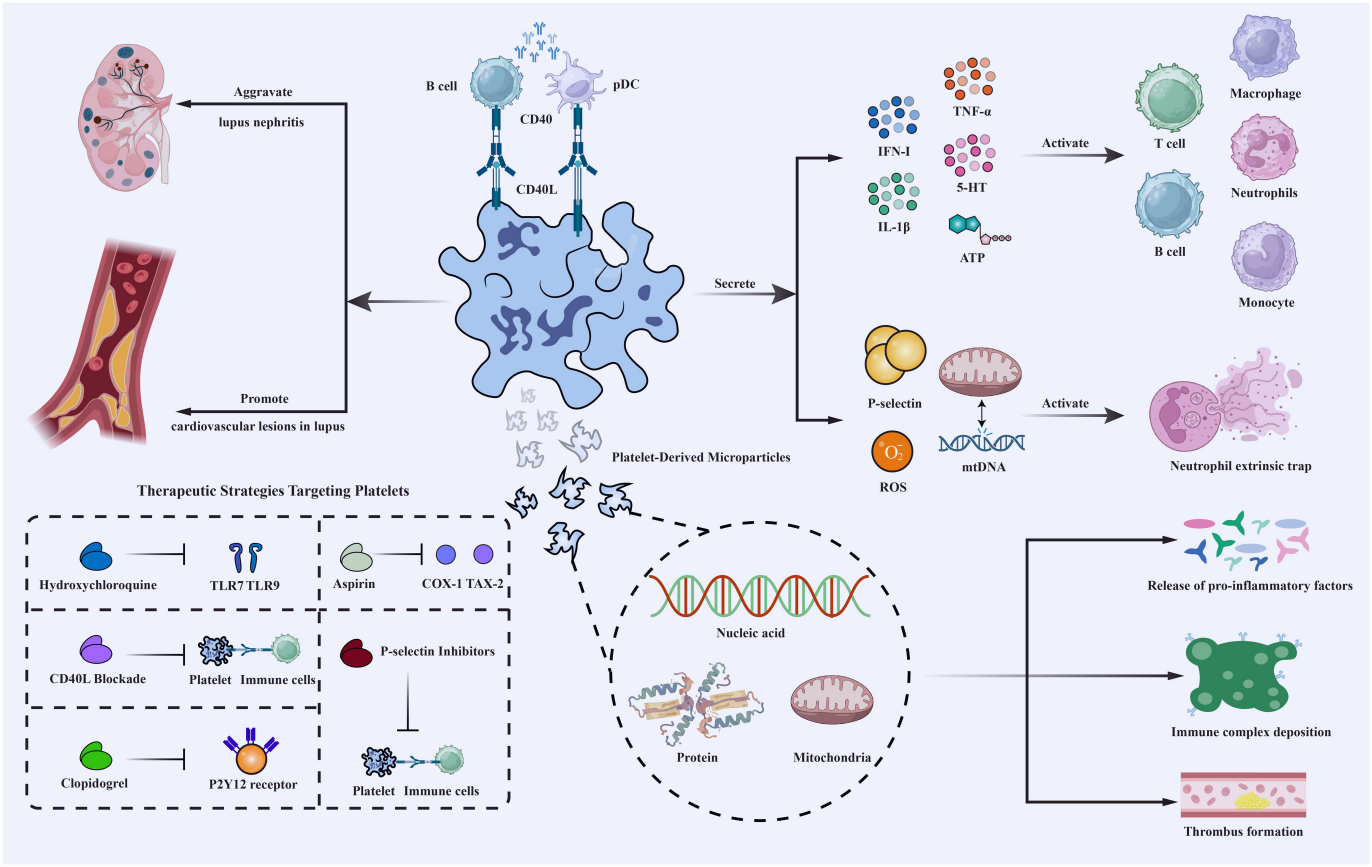
Platelet-driven immune activation and organ injury in SLE. Platelets activate B cells, pDCs, and other immune cells through CD40L–CD40 signaling and by releasing mediators such as TNF-α, IL-1β, 5-HT, PF4, and ATP. Platelets also respond to and amplify type I interferon-related signaling, thereby reinforcing inflammatory immune-cell activation. Platelet-derived microparticles carrying nucleic acids/mtDNA and proteins, together with P-selectin and ROS, promote NET formation, pro-inflammatory factor release, immune-complex deposition, and thrombosis, exacerbating lupus nephritis and cardiovascular lesions. Lower panel: platelet-targeted strategies (hydroxychloroquine, aspirin, clopidogrel, anti-CD40L, P-selectin inhibitors).

In recent years, research has expanded beyond the classical hemostatic role of platelets, uncovering their contributions to immune regulation, inflammation, and tumor metastasis. These findings highlight their broad involvement in physiological and pathological processes not traditionally linked to coagulation (13–17). In autoimmune diseases such as SLE, platelets not only contribute to immunopathology but also display abnormal patterns of activation (18, 19). Excessive platelet activation in SLE is often unnecessary and can be detrimental (10, 20–22). Such dysregulated activity not only amplifies systemic inflammation but also contributes to severe complications, including cardiovascular inflammation and renal fibrosis (23–25). Beyond their classical role in hemostasis, platelets have gained attention as accessible biomarkers. The platelet-to-lymphocyte ratio has been investigated as an inexpensive inflammatory index in SLE and has been associated with disease activity and renal involvement, including lupus nephritis (26). Key platelet-related biomarkers and functional markers in SLE are summarized in **Table 1**. These observations underscore that platelets are not merely passive bystanders but active participants in disease pathogenesis. Their involvement in immune dysregulation and tissue injury highlights them as promising therapeutic targets. Emerging strategies include inhibiting platelet activation pathways—such as P-selectin and CD40L signaling—or modulating platelet-derived pro-inflammatory mediators, with the goal of mitigating inflammation and reducing organ damage (27, 28). SLE is also an important autoimmune background for secondary, or SLE-associated, antiphospholipid syndrome (APS), in which persistent antiphospholipid antibodies contribute to arterial, venous, microvascular, and pregnancy-related thrombotic complications. This association provides an additional clinical context in which platelet activation is closely linked to thrombo-inflammatory damage in SLE. This review therefore provides a comprehensive overview of current evidence on platelet dysfunction in SLE and explores potential therapeutic approaches aimed at modulating platelet activity. An overview of platelet-mediated immune activation, organ injury, and potential platelet-targeted therapeutic strategies in SLE is provided in **Figure 1**.

**Table 1:** Platelet-related biomarkers and functional markers in SLE.

Functional category	Biomarker/marker	Main pathological relevance in SLE	Potential clinical implication
Hematologic inflammatory index	Platelet-to-lymphocyte ratio	Reflects platelet-related inflammation and lymphocyte alteration	May help evaluate SLE activity and lupus nephritis risk
Interferon-related platelet activation	Platelet IFN-I-regulated genes and proteins	Indicates type I interferon-skewed platelet activation	May reflect immune activation and inflammatory amplification
Platelet activation marker	P-selectin	Promotes platelet–leukocyte adhesion, neutrophil activation, NET formation, and vascular inflammation	May indicate disease activity, endothelial injury, and thrombotic tendency
Immune-interaction marker	Platelet CD40L/soluble CD40L	Promotes B-cell activation, immunoglobulin class switching, dendritic cell maturation, and T-cell polarization	May reflect platelet-mediated immune activation and autoantibody amplification
Procoagulant membrane marker	Phosphatidylserine exposure	Supports PMP formation, coagulation activation, and recognition by antiphospholipid or antiplatelet antibodies	May be associated with immune-mediated platelet injury and thrombosis
Platelet surface glycoprotein marker	GPIIb/IIIa conformational change	Exposes neoepitopes and contributes to platelet activation and immune recognition	May reflect platelet activation and platelet autoantigen exposure
Extracellular vesicle marker	Platelet-derived microparticles	Carry inflammatory mediators, nucleic acids, mitochondrial proteins, immunoglobulins, and platelet glycoproteins	May reflect disease activity, immune-complex formation, vascular injury, and thrombosis
PMP cargo markers	PMP-associated mtDNA/oxidized mtDNA, IgG, acetylated histones	Activate nucleic-acid sensing pathways, promote monocyte activation, and enhance NET formation	May indicate nucleic-acid-driven inflammation, immune-complex activity, and organ damage
Platelet–immune cell interaction marker	Platelet–leukocyte aggregates, including platelet–neutrophil aggregates	Promote ROS production, NET release, procoagulant activity, and glomerular inflammation	May indicate thrombo-inflammation, cardiovascular risk, and lupus nephritis progression
Complement-related platelet marker	Platelet-associated C4d	Enhances platelet aggregation and platelet interactions with monocytes and endothelial cells	May indicate complement-mediated vascular inflammation and thrombosis
Immune-complex receptor marker	Platelet FcγRIIA	Mediates immune-complex-induced platelet activation, degranulation, and renal microthrombosis	May be linked to lupus nephritis severity and thrombotic complications
Platelet-derived soluble mediators	PF4, 5-HT, ATP, thromboxane A_2_	Amplify inflammation, immune-cell recruitment, platelet aggregation, and vasoconstriction	May be related to inflammatory activity, thrombosis, and vasomotor dysfunction

**Table 1** summarizes platelet-related biomarkers and functional markers according to their biological relevance and potential clinical implications. Quantitative diagnostic parameters, including sensitivity, specificity, and cut-off values, are not included as separate columns because most platelet-derived markers have not yet been validated as standalone clinical tests in SLE. Among the listed markers, PLR is the most clinically accessible because it is derived from routine blood counts; however, reported cut-off values and diagnostic performance vary substantially across cohorts. For platelet activation markers such as P-selectin and sCD40L, and for extracellular vesicle-based markers such as PMPs, validated SLE-specific cut-offs are not available. Their interpretation is affected by disease activity, lupus nephritis, infection, antiphospholipid antibody status, thrombocytopenia, renal impairment, cardiovascular comorbidities, and treatment exposure. Pre-analytical factors are especially important for PMPs and activation markers, including anticoagulant type, venipuncture technique, time to processing, centrifugation protocol, platelet-poor or platelet-depleted plasma preparation, storage conditions, freeze–thaw cycles, flow-cytometer sensitivity, calibration strategy, and gating method. Therefore, these biomarkers should currently be interpreted as complementary research or risk-stratification tools rather than standalone diagnostic or monitoring tests.

Thrombocytopenia represents another clinically important dimension of platelet involvement in SLE. As one of the common hematological manifestations of lupus, thrombocytopenia may arise from immune-mediated peripheral platelet destruction, antiplatelet antibodies, antiphospholipid antibody-associated platelet activation and consumption, complement deposition on platelet surfaces, and impaired thrombopoiesis. Clinically, it is relevant not only because of bleeding risk but also because it may reflect active systemic autoimmunity and has been associated with disease severity and adverse outcomes. Importantly, thrombocytopenia and platelet activation are not mutually exclusive in SLE. In some patients, particularly those with antiphospholipid antibodies, reduced platelet counts may coexist with platelet hyperreactivity and thrombotic risk. Therefore, platelet biology in SLE should be understood as a spectrum ranging from immune-mediated platelet destruction to pathological platelet activation and thrombo-inflammatory organ damage.

## 2 The pathogenic role of platelets in SLE

### 2.1 Promoting inflammation

#### 2.1.1 Release of pro-inflammatory mediators

Although platelets are anucleate, they retain megakaryocyte-derived RNAs and translational machinery. Platelets from patients with SLE exhibit an enhanced type I interferon-related transcriptomic and proteomic signature, particularly in association with vascular disease. Separately, experimental studies have demonstrated that platelets can process and present antigen-derived peptides to CD8^+^ T cells through MHC class I and promote T-cell activation, including IL-2 and IFN-γ production (29). However, the contribution of this mechanism to human SLE remains uncertain (30–32).

Platelets can contribute to IL-1β production and amplify NLRP3 inflammasome activation in innate immune cells (33, 34). Once secreted, IL-1β acts on endothelial and immune cells, activating NF-κB-related inflammatory signaling and promoting leukocyte recruitment (35). Moreover, megakaryocytes transcribe tumor necrosis factor-α (TNF-α) mRNA before releasing platelets; this mRNA is retained in platelets and translated into protein upon activation (36). The release of TNF-α further amplifies local immune responses and contributes to vascular injury in SLE.

Platelet-derived serotonin 5-HT also contributes to inflammation by activating various immune cells. When 5-HT binds to receptors on T cells, it stimulates the extracellular signal-regulated kinase signaling pathway, which promotes T-cell proliferation and activation (37, 38). Similarly, 5-HT interacts with 5-HT_2_A receptors on neutrophils, inducing chemotaxis and enhancing their directed migration to inflamed tissues. During this process, neutrophils release ROS, which damage cell membranes and DNA, thereby amplifying local inflammation (39, 40). In addition, 5-HT facilitates pathogen recognition by monocytes and activates intracellular signaling pathways, leading to the release of pro-inflammatory cytokines such as TNF-α, IL-1β, and IL-6 (39, 41).

Platelets also release platelet factor 4 (PF4), a key chemokine with potent immunomodulatory functions (42). Once secreted into the extracellular space, PF4 rapidly binds to target cells and signaling molecules. It modulates the recruitment and activation of monocytes, neutrophils, and lymphocytes through chemokine-receptor-dependent pathways (43). PF4 can also directly modulate platelet signaling and immune-cell activation, thereby reinforcing thrombo-inflammatory responses (44).

Platelet dense granules store large amounts of adenosine triphosphate (ATP). Upon platelet activation, injury, or apoptosis, ATP is released into the extracellular space, where it acts as a “danger signal” to initiate immune responses (45). Extracellular ATP binds to P2X7 receptors on platelets and immune cells, promoting calcium and sodium influx across the cell membrane (46). Prolonged activation of these receptors leads to the formation of large transmembrane pores, increasing membrane permeability and ultimately triggering pyroptotic cell death. This process is accompanied by the release of pro-inflammatory cytokines, including IL-1β and IL-18, which further amplify both local and systemic inflammatory responses (47).

In autoimmune diseases such as SLE, platelets can bind, internalize, and transport extracellular DNA, histones, and other nuclear components released from damaged, apoptotic, or NET-forming cells. These platelet-associated nuclear materials may subsequently be carried on platelet surfaces or incorporated into platelet-derived extracellular vesicles. Because platelets lack a nucleus, their own nucleic acid-related inflammatory cargo is derived primarily from mitochondria and megakaryocyte-derived RNA rather than from platelet nuclear material (48). In SLE, extracellular DNA and histones are recognized as damage-associated molecular patterns (DAMPs) and targeted by autoantibodies, particularly anti–double-stranded DNA and anti-histone antibodies. The resulting immune complexes can deposit within glomerular structures, activate complement, and promote inflammatory renal injury in lupus nephritis (49). Mitochondrial DNA (mtDNA) released from platelets represents another key DAMP involved in autoimmune and inflammatory responses (24, 50). Unlike nuclear DNA, mtDNA has structural similarities to bacterial DNA, making it highly immunostimulatory once exposed extracellularly. Extracellular mtDNA can activate nucleic-acid-sensing pathways, including TLR9- and cGAS–STING-related pathways, depending on the responding cell type, thereby promoting type I interferon and inflammatory responses (51, 52). In addition to DNA and histones, activated or dying platelets release RNA and ribonucleoprotein complexes (53, 54). These nucleic acid–protein assemblies are internalized by dendritic cells or macrophages through endocytosis and detected by TLR7/8. This recognition triggers MyD88-dependent signaling, which promotes the production of IFN-I, such as IFN-α, along with other pro-inflammatory cytokines (55–57).

#### 2.1.2 Promotion of neutrophil extracellular traps

Platelets have been shown to promote the formation of neutrophil extracellular traps (NETs) (58, 59). NETs are web-like structures released by neutrophils and consist mainly of decondensed chromatin bound to DNA, histones, and various granule proteins (60). Although initially recognized as a defense mechanism against pathogens, NETs can have detrimental effects in autoimmune diseases such as SLE, where they exacerbate immune activation and tissue damage (61–63). By releasing chromatin, neutrophils expose nuclear components that are normally sequestered within the cell, thereby generating potent autoantigens that trigger and amplify autoimmune responses in SLE (64). Platelets facilitate this process through the surface expression of P-selectin. Upon activation, P-selectin is translocated from α-granules to the platelet membrane, where it binds to its counter-receptor, P-selectin glycoprotein ligand-1 (PSGL-1), on neutrophils. This interaction not only enhances neutrophil activation but also provides a critical signal that initiates NETs (58, 59). Activated platelets release platelet-derived microparticles (PMPs), which are small vesicles enriched with pro-inflammatory mediators such as mtDNA, ROS, and various cytokines (65, 66). PMPs can directly trigger NET formation, thereby amplifying inflammation in SLE (18). Extracellular mitochondrial material can activate TLR9-dependent signaling in neutrophils and promote NET formation. Platelet-derived mitochondrial cargo may therefore contribute to this process in SLE, although the relative importance of oxidized platelet mtDNA requires further clarification (67). In parallel, platelets themselves express TLRs, notably TLR4 and TLR7, which contribute to NETosis. Their activation enhances neutrophil responses, while PMPs carrying TLR ligands can reinforce this process through a positive feedback loop (68, 69). Furthermore, platelet stimulation induces robust ROS production in neutrophils, a critical step in NET formation. ROS activate signaling pathways such as protein kinase C and NADPH oxidase, leading to chromatin decondensation and the subsequent release of NETs (70, 71).

#### 2.1.3 CD40L-CD40 interaction on platelets

CD40 is a key receptor expressed on B cells, dendritic cells, macrophages, and other antigen-presenting cells, whereas its ligand, CD40L (also known as CD154), is typically found on activated T helper cells. The CD40–CD40L interaction is an important costimulatory pathway in SLE and contributes to B-cell activation, autoantibody production, and tissue inflammation (72, 73).

Although CD40L was traditionally considered to originate mainly from activated T cells, recent studies have identified platelets as an additional and significant source. Following activation, platelets upregulate CD40L on their surface and release its soluble form into the circulation (74). In patients with SLE, plasma sCD40L levels are markedly elevated and positively correlate with disease activity (75, 76). Engagement of CD40 by CD40L activates multiple immune cell subsets, promotes their maturation, and enhances their antigen-presenting capacity. In particular, CD40L–CD40 signaling in B cells drives proliferation, supports immunoglobulin class switching, and promotes the production of pathogenic IgG autoantibodies (77). This signaling cascade further amplifies autoantibody production. Beyond B-cell activation, the CD40/CD40L axis also regulates dendritic cell maturation by upregulating major MHC molecules and costimulatory proteins such as CD80 and CD86, thereby enhancing antigen presentation (78). In parallel, CD40/CD40L engagement promotes the differentiation of monocytes into pro-inflammatory M1 macrophages, which release high levels of cytokines and contribute directly to tissue injury through the production of ROS (79, 80). Additionally, CD40L expressed on the platelet surface drives the polarization of CD4^+^ T cells toward pathogenic Th1 and Th17 subsets, while simultaneously impairing the expansion and function of regulatory T cells. This imbalance further compromises immune tolerance and fuels the progression of autoimmunity in SLE (72, 81).

#### 2.1.4 Platelets as autoantigens

Recent studies indicate that platelets themselves can act as autoantigens in SLE, thereby initiating autoimmune responses (82). During platelet activation or injury, substantial changes occur in the composition of the platelet membrane, particularly in its phospholipid distribution. Under physiological conditions, anionic phospholipids such as phosphatidylserine (PS) are confined to the inner leaflet of the membrane and remain concealed from immune recognition. During strong platelet activation or apoptosis, phosphatidylserine is externalized to the outer membrane leaflet, creating a procoagulant surface and potentially exposing phospholipid-associated antigenic structures.

In addition to phospholipid exposure, platelet activation alters the surface availability and activation state of glycoproteins such as GPIIb/IIIa, which may facilitate antibody binding or immune recognition in susceptible individuals. As a result, activated or damaged platelets may be misidentified as “non-self” and targeted by antibody-mediated immune responses (18). Severe platelet activation, injury, or apoptosis can promote the release of mitochondria, mitochondrial DNA, megakaryocyte-derived RNAs, and platelet-derived extracellular vesicles. Platelets may also carry extracellular DNA, histones, and nucleoprotein complexes originating from other damaged or dying cells. These platelet-associated or platelet-transported materials may enhance nucleic acid sensing, immune-complex formation, and autoantibody-mediated inflammation in SLE (83, 84). Complement components such as C3b and C4b can deposit directly onto platelet surfaces, marking them for immune recognition and clearance (85–87). Moreover, specific autoantibodies, including antiplatelet and antiphospholipid antibodies, can bind to platelet surface phospholipids and glycoproteins. Through mechanisms such as cross-reactivity and molecular mimicry, these autoantibodies promote immune-mediated platelet injury and contribute to disease progression (88, 89). These mechanisms provide a pathological basis for SLE-associated thrombocytopenia. Antibody-coated platelets can be cleared by splenic and hepatic macrophages through Fcγ receptor-dependent phagocytosis, while complement deposition may further enhance platelet opsonization or directly promote platelet injury. In addition to accelerated peripheral destruction, impaired platelet production may also contribute to thrombocytopenia in SLE, particularly when autoantibodies, inflammatory cytokines, or type I interferon-related immune activation affect megakaryocyte maturation and thrombopoiesis. Thus, thrombocytopenia in SLE reflects a combined process involving platelet autoantigen exposure, immune-mediated clearance, complement activation, and defective platelet generation.

### 2.2 Platelet-derived microparticles

#### 2.2.1 Characteristics of platelet-derived microparticles

PMPs were first identified by Peter Wolf in 1967 as platelet fragments involved in blood coagulation (90). Later studies established that PMPs are extracellular vesicles released from activated platelets. Their formation begins with platelet activation, which can be triggered by soluble agonists, mechanical shear stress, or signaling through membrane glycoproteins such as GPIIb/IIIa (65). During activation, platelets undergo membrane remodeling, leading to the translocation of PS and other anionic phospholipids from the inner to the outer leaflet of the plasma membrane. The exposure of PS is a hallmark of PMP formation, as it promotes both the generation and release of these vesicles (91, 92).

PMPs typically range from 100 to 1000 nm in diameter, placing them within the category of extracellular vesicles. Compared with exosomes (30–150 nm), PMPs differ in size, density, morphology, and biogenesis. Platelet-derived microvesicles are generally generated by outward budding and shedding of the plasma membrane, whereas exosome-enriched vesicles originate from the endosomal multivesicular-body pathway and are released following fusion with the plasma membrane (65, 93). The membranes of PMPs retain markers derived from their parent platelets, including CD41, CD42b, and CD61. A variable proportion of PMPs also expose phosphatidylserine, particularly when they are generated during strong platelet activation or apoptosis. Because phosphatidylserine exposure is not specific to a single extracellular-vesicle subtype, platelet-associated markers and standardized detection strategies are required for PMP identification (94, 95). Proteomic studies further suggest that PMP size influences molecular cargo: smaller PMPs (100–500 nm) are enriched in α-granule proteins, while larger PMPs contain higher levels of lipid mediators and mitochondrial proteins (96, 97). PMPs can also encapsulate intact mitochondria, mtDNA, and nucleic acids such as microRNAs, all of which contribute to their proinflammatory activity​ (98). In SLE, PMP composition is altered. For example, PMPs from SLE patients have been shown to carry glycolytic enzymes, mitochondrial proteins, and immunoglobulins. Moreover, acetylated histones have been detected in SLE-derived PMPs, where they may promote NET formation and exacerbate tissue damage (48, 93, 99).

#### 2.2.2 Pathogenic role of PMPs in SLE

Altered circulating levels and molecular profiles of PMPs have been reported in patients with SLE. Some PMP subsets, particularly those carrying immunoglobulins, complement components, or nucleic acid-related cargo, have been associated with disease activity and organ involvement. However, these associations vary across studies because of differences in patient populations, sample processing, PMP definitions, and detection methods. PMPs enhance inflammatory responses through several pathways. Their high expression of CD41 facilitates uptake by neutrophils, which then release leukotrienes and the proinflammatory cytokine IL-1β. These mediators stimulate fibroblasts to secrete IL-6 and IL-8, thereby promoting joint and tissue inflammation (93). In addition, IgG-bearing PMPs can bind to and be internalized by monocytes from patients with SLE. *In vitro*, IgG-opsonized PMPs increase monocyte expression of CD69 and CD64 and stimulate the production of IL-1β, TNF-α, and IFN-α, further exacerbating immune dysregulation (100, 101). Moreover, PMPs serve as key carriers of autoantigens and actively contribute to immune complex formation and deposition. PMPs can carry multiple immunologically active molecules, including nucleic acids, HMGB1, immunoglobulins, and platelet glycoproteins such as GPIIIa. Nucleic acid-containing PMPs may interact with anti-dsDNA antibodies and participate in the formation of circulating immune complexes. In contrast, HMGB1 and platelet glycoproteins may contribute independently to innate immune activation, autoantigen exposure, and platelet-directed immune responses. Together, these processes may promote vascular inflammation and tissue injury in SLE (102, 103).

PMPs amplify inflammatory cytokine production by interacting with leukocytes, particularly monocytes and neutrophils (104). Both platelet–leukocyte aggregates and the PMPs they release stimulate monocytes to produce reactive oxygen species and adopt a procoagulant phenotype, thereby promoting thrombosis (105, 106). Clinically, elevated levels of PS^+^ PMPs in patients with SLE are strongly associated with a higher incidence of thrombotic events. In addition, P-selectin expression may serve as a marker of disease activity. This cascade not only intensifies intravascular inflammation and endothelial injury but also contributes to the elevated risk of cardiovascular complications in SLE. Platelet-derived membrane vesicles can deliver CD40L/CD154 to B cells and promote antigen-specific humoral responses, including IgG production, in experimental models (107). In SLE, platelet–B-cell interactions have also been associated with B-cell activation and circulating immunoglobulin levels, although the specific contribution of PMPs requires further study (107). In addition, miRNAs carried by PMPs can be internalized by endothelial and immune cells, where they modulate gene expression and provide a molecular basis for PMP-mediated intercellular inflammatory signaling in SLE.

### 2.3 Platelets and lupus nephritis

In SLE, platelets play a central role in renal injury. FcγRIIA, the only immune complex receptor expressed on human platelets, triggers platelet degranulation upon activation, releasing large amounts of pro-inflammatory mediators and extracellular vesicles that recruit and activate neutrophils. Studies have shown that FcγRIIA-mediated platelet activation amplifies type I interferon-related inflammation and promotes immune-complex deposition along glomerular capillary walls and within the mesangium. The resulting complement activation and inflammatory-cell recruitment contribute to glomerular basement-membrane injury, mesangial expansion, and endocapillary proliferation. Furthermore, FcγRIIA-driven platelet activation markedly facilitates renal microthrombus formation, causing local ischemia and tissue damage (24). Experimental studies show that FcγRIIA-transgenic mice develop renal manifestations—including proteinuria, endothelial injury, and interstitial nephritis—earlier and more severely than non-transgenic controls (23). Moreover, several reports demonstrate that targeting platelets in immune complex–mediated injury models and lupus-prone mice significantly ameliorates proliferative nephropathy (108, 109).

In SLE, activation of platelet TLR7 promotes platelet activation and facilitates the formation of platelet–low-density neutrophil aggregates. The physical interaction between platelets and neutrophils is likely mediated by adhesion pathways such as P-selectin–PSGL-1, whereas TLR7 signaling enhances platelet responsiveness and promotes NET formation. These platelet–neutrophil interactions may contribute to renal inflammation and lupus nephritis. The DNA, RNA, and pro-inflammatory proteins released from NETs exacerbate glomerular basement membrane injury and activate IFN-I pathways, thereby amplifying the inflammatory response (71, 110). Low-density neutrophils also exhibit an immature, pro-inflammatory phenotype and directly damage renal structures by releasing granule enzymes and inflammatory mediators. Studies have shown that platelet–neutrophil aggregates infiltrate glomerular regions extensively, intensify immune complex deposition and complement activation, and ultimately drive glomerulosclerosis and renal fibrosis (68).

In patients with SLE, activated platelets induce CD40 expression in mesangial cells through a CD154-dependent, contact-mediated mechanism (111). This effect requires direct platelet–mesangial contact and can be blocked by anti-CD154 antibodies. Increased CD40 expression on mesangial cells is strongly associated with functional disturbances, including elevated production of monocyte chemoattractant protein-1 and ROS, both of which intensify glomerular immune-inflammatory responses (112). Histological analyses have revealed abundant CD40-expressing mesangial cells within the glomeruli of patients with lupus nephritis, correlating with the severity of inflammation and fibrosis. Through this pathway, platelets not only drive mesangial cell proliferation but also enhance the secretion of transforming growth factor-β1 (TGF-β1), a profibrotic cytokine strongly implicated in glomerulosclerosis (113). In addition, activated platelets in SLE release multiple pro-inflammatory mediators—including platelet-derived growth factor, PF4, and RANTES—that further amplify glomerular inflammation and worsen tissue injury (48). Platelet activation is also linked to microvascular thrombosis, which may cause ischemic damage and interstitial fibrosis in the kidneys.

### 2.4 Involvement in cardiovascular diseases

#### 2.4.1 Involvement in thrombosis

Under normal physiological conditions, platelet activation is an essential defense mechanism that promotes blood coagulation and accelerates vascular repair following injury (114, 115). However, excessive or uncontrolled activation can result in abnormal thrombus formation. In SLE, anti–double-stranded DNA (dsDNA) antibodies circulate either freely or bound to dsDNA, forming immune complexes (116, 117). These complexes have a strong affinity for FcγRIIA receptors on platelet surfaces, directly engaging them and inducing persistent platelet activation (118, 119). In this sustained hyperactive state, platelets continuously release procoagulant factors, thereby amplifying the coagulation cascade.

In addition to anti-dsDNA-containing immune complexes, antiphospholipid antibodies provide a major mechanism linking SLE to platelet-driven thrombosis. SLE-associated antiphospholipid syndrome represents a prototypical thrombo-inflammatory condition in which aPL antibodies, platelet hyperreactivity, complement activation, endothelial injury, and coagulation activation converge. Antiphospholipid antibodies, particularly anti-β2-glycoprotein I antibodies, can promote platelet activation through β2GPI-dependent mechanisms. Multivalent anti-β2GPI–β2GPI complexes engage platelet glycoprotein Ibα and apolipoprotein E receptor 2 (ApoER2), activate intracellular signaling pathways, and enhance platelet responsiveness (120). FcγRIIA may contribute to platelet activation by IgG-containing immune complexes, but it should be distinguished from the GPIbα/ApoER2-dependent β2GPI pathway. Activated platelets upregulate P-selectin and CD40L, activate GPIIb/IIIa, release thromboxane A_2_, expose phosphatidylserine, and shed platelet-derived microparticles. These microparticles provide phosphatidylserine-rich catalytic surfaces for thrombin generation and also amplify platelet–leukocyte and platelet–endothelial interactions.

aPL-mediated thrombosis further depends on cooperation between platelets, complement, and tissue factor pathways. Antiphospholipid antibodies can activate complement, and complement-derived mediators such as C5a promote tissue factor expression and procoagulant activity in monocytes and neutrophils. Activated endothelial cells and leukocytes also express tissue factor, initiating the extrinsic coagulation pathway. Platelets amplify this process by forming platelet–monocyte and platelet–neutrophil aggregates through P-selectin/PSGL-1 and CD40L-mediated interactions, by exposing phosphatidylserine-rich membranes, and by interacting with tissue factor-bearing leukocyte-derived microparticles. Collectively, these interactions accelerate thrombin generation, fibrin formation, and vascular occlusion, thereby linking antiphospholipid antibodies, platelet hyperreactivity, complement activation, tissue factor expression, and arterial, venous, or microvascular thrombosis in SLE-associated APS. These processes accelerate thrombin generation and fibrin formation, thereby linking antiphospholipid antibodies, platelet hyperreactivity, tissue factor upregulation, and thrombosis in SLE-associated APS.

In addition, platelets interact with the complement system, recruiting complement components to sites of vascular injury. Dysregulated complement activation further promotes thrombosis (121). For instance, localized activation of complement can insert sublytic levels of C5b-9 into platelet membranes, generating procoagulant microparticles and amplifying platelet activation (122). The C3a–C3aR axis also facilitates platelet adhesion and spreading (123). Moreover, deposition of C4d on platelets not only enhances platelet aggregation but also fosters interactions among platelets, monocytes, and endothelial cells, thereby intensifying vascular inflammation and creating a prothrombotic environment (37).

#### 2.4.2 Involvement in atherosclerosis

Platelets contribute significantly to both the initiation and progression of atherosclerosis. Dysregulation of the immune system promotes excessive platelet activation, leading to the release of pro-inflammatory mediators and reactive oxygen species (ROS), which can directly injure endothelial cells (124–126). Endothelial damage compromises vascular barrier integrity, facilitating lipid deposition and inflammatory cell infiltration into the vessel wall (127). Once low-density lipoprotein (LDL) penetrates the endothelium, it undergoes oxidative modification to form oxidized LDL, a key driver of atherosclerotic plaque development (128–131).

Through interactions with immune cells, platelets can further activate these cells and perpetuate chronic inflammation (32). In patients with SLE, platelet binding to immune cells promotes their aggregation within the vessel wall, leading to the release of additional inflammatory mediators and the establishment of a self-amplifying inflammatory loop. Platelet-derived mediators and platelet–macrophage interactions may enhance macrophage recruitment, inflammatory activation, and uptake of modified lipoproteins, thereby promoting foam-cell formation. The accumulation of lipid-laden macrophages and other vascular cell types contributes to the development of the lipid-rich plaque core (132–134). Importantly, foam cells arise not only from macrophages but also from vascular smooth muscle cells, endothelial cells, and stem cell–derived lineages. The progressive buildup of these cells contributes both to plaque expansion and to the destabilization that predisposes plaques to rupture and subsequent thrombus formation.

Interactions between platelets and endothelial cells represent a key mechanism in the pathogenesis of vascular disease among patients with SLE (135, 136). Platelets from SLE patients strongly stimulate human umbilical vein endothelial cells, leading to increased expression of pro-inflammatory genes such as interleukin-8 (IL-8), vascular cell adhesion molecule-1, and intercellular adhesion molecule-1 (137). The upregulation of these molecules enhances endothelial adhesiveness and promotes inflammatory responses, thereby accelerating the progression of atherosclerosis and increasing the risk of cardiovascular complications in SLE (32).

#### 2.4.3 Involvement in vasomotor disorders

Platelets may also contribute to vasomotor abnormalities in SLE, with Raynaud’s phenomenon representing the most clinically recognizable correlate. Raynaud’s phenomenon is characterized by episodic vasospasm of digital arteries and cutaneous arterioles, typically triggered by cold exposure or emotional stress. Clinically, this manifests as transient digital ischemia with pallor, cyanosis, and reperfusion-associated erythema, often accompanied by numbness, pain, or paresthesia (138). In patients with SLE, such attacks may reflect an imbalance between platelet-derived vasoconstrictive mediators and endothelial vasodilatory responses (139).

Platelets are the major circulating reservoir of 5-hydroxytryptamine (5-HT). Upon activation, platelet-derived 5-HT can directly stimulate receptors on vascular smooth muscle cells, thereby promoting vasoconstriction. Under physiological conditions, this constrictive effect is counterbalanced by endothelial release of nitric oxide (NO) and prostacyclin (140). However, in SLE, chronic inflammation, immune-complex deposition, oxidative stress, and endothelial dysfunction may weaken these compensatory vasodilatory pathways. As a result, platelet-derived 5-HT may exert a more pronounced vasoconstrictive effect, predisposing the digital microcirculation to exaggerated vasospasm (141).

Activated platelets also release thromboxane A_2_, another potent vasoconstrictor and platelet agonist. Thromboxane A_2_ further enhances platelet aggregation and vascular smooth muscle contraction, while also interfering with endothelial NO-dependent relaxation. Together, 5-HT and thromboxane A_2_ create a feed-forward loop in which platelet activation promotes vasoconstriction, reduced blood flow promotes endothelial stress, and endothelial injury further amplifies platelet activation (142, 143). This mechanism provides a plausible link between platelet hyperactivity and the cold- or stress-induced digital color changes observed in SLE-associated Raynaud’s phenomenon.

### 2.5 Platelet-derived biomarkers in SLE

Platelet-related biomarkers may provide a translational bridge between platelet pathobiology and clinical assessment in SLE. These markers can be broadly divided into hematologic indices, platelet activation markers, extracellular vesicle-based markers, platelet–immune cell interaction markers, and platelet-associated complement markers. PLR is the most accessible marker because it is calculated from routine blood counts and has been associated with SLE activity and lupus nephritis in several clinical studies (26). However, PLR is not platelet-specific and may be influenced by lymphopenia, thrombocytopenia, infection, glucocorticoid exposure, immunosuppressive therapy, renal involvement, and systemic inflammation. Therefore, PLR is better regarded as an inexpensive adjunctive inflammatory index rather than a standalone diagnostic marker.

Platelet activation markers, including P-selectin and sCD40L, may more directly reflect platelet-mediated immune activation. P-selectin is closely related to platelet–leukocyte adhesion, NET formation, endothelial activation, and thrombotic tendency, while sCD40L reflects the CD40L–CD40 immune axis involved in B-cell activation, dendritic-cell maturation, and vascular inflammation (38, 75, 76). Nevertheless, these markers are highly sensitive to pre-analytical conditions. Ex vivo platelet activation during venipuncture, delayed processing, inappropriate anticoagulant selection, sample agitation, and freeze–thaw cycles may artificially alter measured levels. In addition, soluble P-selectin can be derived from endothelial cells, and serum sCD40L may be increased during clot formation, limiting platelet specificity.

PMPs and PMP-associated cargoes are particularly attractive biomarkers because they can carry phosphatidylserine, platelet glycoproteins, mitochondrial DNA, oxidized mtDNA, immunoglobulins, acetylated histones, and complement-related molecules (4, 99). These features link PMPs to immune-complex formation, nucleic-acid sensing, NET formation, vascular injury, thrombosis, and lupus nephritis. However, PMP detection remains technically challenging. Differences in centrifugation protocols, platelet-poor versus platelet-depleted plasma preparation, storage conditions, freeze–thaw history, antibody panels, flow-cytometer sensitivity, size calibration, and gating strategies can lead to substantial inter-laboratory variability. Thus, although PMPs are mechanistically informative, their routine clinical use requires further assay standardization and prospective validation.

Other markers, such as platelet-associated C4d, platelet–leukocyte aggregates, FcγRIIA-related platelet activation, and platelet-derived soluble mediators, may help reflect complement activation, thrombo-inflammation, immune-complex-mediated platelet activation, or vasomotor dysfunction (85–87). However, their diagnostic, prognostic, and monitoring value remains incompletely defined. Overall, no platelet-derived biomarker has yet been universally accepted as a standalone clinical test for SLE. Future biomarker studies should use standardized sample-processing protocols, report sensitivity, specificity, and cut-off values when applicable, and adjust for disease activity, treatment exposure, infection, renal impairment, antiphospholipid antibody status, thrombocytopenia, and cardiovascular comorbidities.

## 3 Therapeutic strategies targeting platelets

### 3.1 Hydroxychloroquine

Numerous studies have shown that platelet activation is markedly increased in patients with SLE compared with healthy individuals, suggesting that inhibiting excessive platelet activation may be an important therapeutic strategy (25, 48, 144). Hydroxychloroquine (HCQ), a first-line therapy for SLE, not only modulates immune function but also exerts multiple inhibitory effects on platelet activation. Specifically, HCQ may attenuate endosomal TLR7/9-related signaling, reduce platelet α-granule secretion, and decrease activation of the integrin αIIbβ3 pathway, thereby limiting platelet adhesion and aggregation (145–149). In addition, HCQ alters the electrostatic distribution of plasma membrane phospholipids, preventing antiphospholipid antibody binding to β2-glycoprotein I and thereby reducing platelet adhesion and aggregation (150–152). Clinical studies support this mechanism, showing that long-term HCQ use is associated with a significantly lower incidence of venous and arterial thrombotic events in patients with SLE (153, 154).

Evidence summary and limitations. Among the platelet-modulating agents discussed in this review, HCQ has the strongest clinical foundation because it is a standard-of-care therapy for SLE and is recommended for most patients unless contraindicated. Its potential antiplatelet and antithrombotic effects are supported by mechanistic studies, ex vivo platelet assays, and observational clinical data, particularly in patients with SLE or aPL positivity. However, the specific contribution of platelet inhibition to the overall clinical benefit of HCQ remains difficult to isolate, because HCQ also modulates innate immune signaling, antigen presentation, cytokine production, and lipid metabolism. Therefore, HCQ should be viewed as a broad immunomodulatory therapy with platelet-inhibitory properties rather than as a selective antiplatelet drug.

### 3.2 CD40L blockade

Anti-CD40L antibodies exert protective effects in SLE by blocking CD40L–CD40 interactions, thereby suppressing the pro-inflammatory and procoagulant functions of platelets. Mechanistically, CD40L inhibition reduces immune complex–induced platelet activation and decreases the release of pro-inflammatory mediators such as PF4 and high-mobility group box 1 protein (155, 156). Blockade of the CD40–CD40L axis can inhibit T-cell-dependent B-cell activation and may attenuate endothelial inflammatory signaling. However, evidence that CD40L blockade directly suppresses platelet-driven NET formation in SLE remains insufficient (157–159). Furthermore, CD40L blockade downregulates vascular cell adhesion molecule-1 and intercellular adhesion molecule-1 expression on endothelial cells, thereby improving microvascular function (160, 161). Clinical studies suggest that anti-CD40L therapy has substantial therapeutic potential in lupus nephritis, including reducing anti-dsDNA antibody production, alleviating glomerular inflammation, and increasing serum complement levels (73).

Evidence summary and limitations. The CD40L–CD40 pathway is strongly supported by mechanistic and translational evidence as a central axis linking platelets, B-cell activation, endothelial inflammation, and lupus nephritis. However, the clinical development of CD40L blockade has been complicated by safety concerns and heterogeneous efficacy signals. Early anti-CD40L antibodies were limited by thromboembolic adverse events, probably related to Fc-dependent platelet activation, whereas newer Fc-silent or Fc-free CD40L inhibitors are being developed to reduce this risk. Thus, CD40L blockade remains a biologically compelling strategy, but its role as a platelet-directed therapy in SLE requires further validation in adequately powered clinical trials with careful thrombotic and bleeding safety monitoring.

### 3.3 Clopidogrel

P2Y purinoceptor 12 (P2Y12) is a key adenosine diphosphate (ADP) receptor expressed on platelets, where it plays a central role in ADP-mediated platelet activation and aggregation (162). Clopidogrel inhibits P2Y12, thereby blocking ADP-induced platelet aggregation and reducing the risk of thrombosis. In addition to suppressing platelet–platelet aggregation, Clopidogrel inhibits ADP-dependent platelet activation through P2Y12 and thereby reduces platelet aggregation (163), processes closely linked to the inflammatory responses in SLE. Clinical studies show that clopidogrel lowers plasma levels of sCD40L in patients with SLE while concurrently inhibiting platelet hyperactivation (164).

Evidence summary and limitations. The evidence supporting clopidogrel in SLE remains limited. Available clinical data are mainly derived from a small exploratory phase I/II pilot study that evaluated pharmacodynamic outcomes, such as soluble CD40L levels and platelet activation markers, rather than hard clinical endpoints including thrombosis, renal survival, flare prevention, or cardiovascular events. Additional supportive evidence comes from lupus-prone mouse models, in which P2Y12 inhibition improved renal manifestations and disease activity. Therefore, clopidogrel should be considered an experimental or individualized platelet-directed strategy in SLE rather than an established disease-modifying treatment. Its routine use for thrombosis prevention in aPL-positive SLE or SLE-associated APS is not currently supported by guideline-level evidence.

### 3.4 Aspirin

Cyclooxygenase-1 is a key enzyme in platelet thromboxane A_2_ synthesis. Thromboxane A_2_ is a potent platelet agonist and vasoconstrictor that plays a central role in thrombogenesis (165, 166). Aspirin irreversibly acetylates the serine residue at the cyclooxygenase-1 active site, thereby blocking the conversion of arachidonic acid to prostaglandin H_2_ and ultimately inhibiting TXA_2_ production (167, 168). Through this mechanism, aspirin markedly reduces platelet activation and aggregation. In SLE patients with high-risk antiphospholipid antibody profiles but no previous thrombotic or obstetric APS, low-dose aspirin may be considered for primary thrombosis prevention according to risk stratification. In patients with established thrombotic APS, antiplatelet therapy is usually considered as an adjunct to anticoagulation in selected high-risk settings rather than as a stand-alone strategy. Therefore, the platelet-inhibitory effect of aspirin is particularly relevant in SLE-associated APS or aPL-positive SLE, but its use should be individualized according to thrombotic history, antibody profile, bleeding risk, and concomitant cardiovascular risk factors (169). In recent years, dual antiplatelet therapy—such as aspirin combined with clopidogrel—has shown greater efficacy in high-risk thrombotic patients, particularly when enhanced platelet inhibition is required (170, 171). Studies have also demonstrated that aspirin reduces levels of sCD40L and soluble P-selectin in healthy individuals (172, 173). However, the specific relevance of this mechanism in SLE remains unclear. These findings underscore the need for further research to clarify the impact of aspirin on immuno-inflammatory markers in SLE and to optimize its therapeutic use.

Evidence summary and limitations. Aspirin has the clearest clinical role in aPL-positive SLE patients with high-risk aPL profiles, particularly for primary thrombosis prevention when bleeding risk is acceptable. Current international recommendations support low-dose aspirin in asymptomatic aPL carriers, patients with SLE without previous thrombotic or obstetric APS, and non-pregnant women with previous obstetric APS only when a high-risk aPL profile is present. However, aspirin should not be generalized to all patients with SLE, and it is not a substitute for anticoagulation in established thrombotic APS. In patients with thrombocytopenia, renal impairment, gastrointestinal bleeding risk, or concomitant anticoagulant therapy, the benefit–risk balance of aspirin requires individualized assessment.

### 3.5 P-selectin inhibitors

P-selectin inhibitors, which block platelet surface P-selectin and its interaction with PSGL-1, have shown therapeutic potential in SLE (174, 175). In SLE, P-selectin upregulation contributes to platelet–leukocyte aggregation, thrombus formation, and the progression of vascular inflammation. By disrupting P-selectin–PSGL-1 binding, these inhibitors reduce adhesion among platelets, leukocytes, and endothelial cells. By reducing platelet–neutrophil adhesion, blockade of the P-selectin–PSGL-1 axis may attenuate NET formation and thrombo-inflammatory signaling, thereby alleviating endothelial inflammation and lowering the risk of thrombosis driven by platelet–endothelial adhesion (176–178). In addition, P-selectin inhibition decreases immune complex–induced platelet activation, reducing the downstream systemic inflammatory response (179, 180). These mechanisms are particularly important for ameliorating SLE-associated microvascular lesions, leading to improved microcirculatory function, reduced renal pathology, decreased proteinuria, and attenuation of inflammation-driven tissue damage. Studies in lupus-prone mice suggest that engagement of PSGL-1 by P- or E-selectin activates Syk-dependent signaling and increases intracellular calcium levels in regulatory T cells, particularly follicular regulatory T cells, thereby impairing their suppressive function. Blocking selectin–PSGL-1 interactions may attenuate this signaling, preserve regulatory T-cell activity, reduce pathogenic autoantibody production, and ameliorate renal injury. These findings support selectin blockade as a potential immunomodulatory strategy, although its effects may not be exclusively platelet dependent (174, 181). Collectively, these findings highlight the multifaceted therapeutic potential of P-selectin inhibitors in SLE and support their continued clinical development.

Evidence summary and limitations. Compared with HCQ or aspirin, the evidence for P-selectin inhibition in SLE remains largely preclinical. Experimental studies support the role of the P-selectin/PSGL-1 axis in platelet–leukocyte aggregation, NET formation, endothelial activation, and lupus-like tissue injury. However, clinical evidence demonstrating that P-selectin blockade improves SLE disease activity, lupus nephritis, or thrombosis outcomes is still insufficient. Moreover, P-selectin may have context-dependent effects on immune-cell trafficking and immune regulation, suggesting that complete pathway inhibition may not be uniformly beneficial. Therefore, P-selectin inhibitors should be described as promising investigational agents rather than clinically established therapies for SLE.

### 3.6 Antiplatelet therapy in aPL-positive SLE and SLE-associated APS

In aPL-positive SLE, platelet-directed therapy should be considered within a risk-stratified clinical framework rather than applied uniformly to all patients. Thrombotic risk is influenced by the aPL profile, previous thrombotic or obstetric APS events, concomitant SLE activity, traditional cardiovascular risk factors, platelet count, and bleeding risk (182, 183). A high-risk aPL profile generally includes lupus anticoagulant positivity, double or triple aPL positivity, or persistently high aPL titers (182). Current international recommendations support low-dose aspirin for primary thrombosis prevention in asymptomatic aPL carriers, patients with SLE without previous thrombotic or obstetric APS, and non-pregnant women with a history of obstetric APS only when they have a high-risk aPL profile and acceptable bleeding risk. In contrast, patients with definite thrombotic APS generally require long-term anticoagulation, usually with vitamin K antagonists, and antiplatelet therapy should not be regarded as an equivalent substitute for anticoagulation (182). In selected high-risk settings, such as arterial thrombosis or recurrent thrombosis despite adequate anticoagulation, low-dose aspirin may be considered as an adjunctive strategy (182, 184).

The evidence base for clopidogrel in aPL-positive SLE or SLE-associated APS is more limited than that for aspirin. Although clopidogrel inhibits ADP-dependent platelet activation through the P2Y12 receptor, the potential clinical value of P2Y12 inhibitors in APS remains insufficiently studied (183). Current guideline-based APS management therefore does not support the routine use of clopidogrel for primary thrombosis prevention (182, 183). It may be considered only on an individualized basis, such as in patients with aspirin intolerance or an independent arterial cardiovascular indication, while recognizing that direct APS-specific evidence is limited. Therefore, aspirin, clopidogrel, and other platelet-directed agents should be selected according to the balance between thrombotic and bleeding risks rather than on the basis of aPL positivity alone (182–184).

## 4 Conclusion

Platelets are not passive bystanders in SLE but active mediators that connect immune dysregulation with thrombo-inflammatory organ damage. Mechanistically, activated platelets amplify inflammation through type I interferon–related activation, CD40L–CD40 signaling, release of ATP, serotonin, PF4, and inflammatory cytokines, and generation of platelet-derived microparticles carrying nucleic acids, mitochondrial DNA, and immune-active proteins. These pathways promote NET formation, immune-complex deposition, endothelial dysfunction, microvascular thrombosis, lupus nephritis, and cardiovascular complications.

The main clinical implication is that platelets may serve as both accessible biomarkers and actionable therapeutic targets in SLE. Platelet-related indices, platelet activation markers, platelet-derived microparticles, and platelet–immune cell aggregates may help reflect disease activity, renal involvement, thrombotic risk, or vascular injury. Therapeutically, agents that modulate platelet activation, including hydroxychloroquine, aspirin, clopidogrel, CD40L blockade, and P-selectin inhibition, may complement conventional immunosuppressive strategies, particularly in patients with thrombo-inflammatory or vascular manifestations.

Several challenges remain before platelet-centered approaches can be fully translated into clinical practice. Current evidence is limited by heterogeneous definitions of platelet activation, non-standardized assays for platelet-derived microparticles, small or cross-sectional cohorts, and confounding effects of disease activity and medication exposure. Future studies should prioritize longitudinal cohorts, standardized and cargo-resolved microparticle detection, functional assays of platelet–immune crosstalk under physiologic shear, and biomarker-guided clinical trials. These efforts may clarify how platelet-targeted strategies can improve renal and cardiovascular outcomes while maintaining an acceptable bleeding safety profile.
